# The Association Between Serum Vancomycin Level and Clinical Outcome in Patients With Peritoneal Dialysis Associated Peritonitis

**DOI:** 10.1016/j.ekir.2023.09.013

**Published:** 2023-09-17

**Authors:** Erin Deacon, Mark Canney, Brendan McCormick, Tim Ramsay, Mohan Biyani, Pierre Antoine Brown, Deborah Zimmerman

**Affiliations:** 1 Faculty of Medicine, University of Ottawa, Ontario, Canada; 2 Department of Medicine, Ottawa Hospital, Faculty of Medicine, University of Ottawa and the Kidney Research Centre of the Ottawa Hospital Research Institute, Ottawa, Ontario, Canada; 3 Ottawa Methods Centre, Ottawa Hospital Research Institute, Ottawa, Ontario, Canada

**Keywords:** intraperitoneal, outcome, peritonitis, peritoneal dialysis, vancomycin

## Abstract

**Introduction:**

Intraperitoneal (IP) vancomycin is often first-line empiric therapy and then maintenance therapy for peritoneal dialysis (PD) peritonitis. However, how vancomycin serum levels correlate with clinical outcomes remains unclear.

**Methods:**

We conducted a retrospective single-center adult cohort study of 98 patients with PD peritonitis treated with IP vancomycin between January 2016 and May 2022. The association between nadir vancomycin level and cure was evaluated in a logistic regression model, first unadjusted and then adjusted for age, sex, weight, glomerular filtration rate (GFR), and total number of days on PD. Vancomycin was assessed both as a continuous exposure (per 1 mg/l increase) and as a categorical exposure (<15 mg/l vs. ≥15 mg/l). A receiver operating characteristic curve (ROC) was created to explore nadir vancomycin level thresholds in an attempt to identify an optimal target level during treatment.

**Results:**

Of the patients, 81% achieved cure, and patients with nadir vancomycin level ≥15 mg/l were 7.5 times more likely to experience cure compared to those with a nadir level <15 mg/l (odds ratio [OR] 7.58, 95% confidence interval [CI] 1.71–33.57, *P* = 0.008). Weight, GFR, days on PD, sex, and age were not independently associated with outcome. The vancomycin level with the greatest discriminatory capacity for cure on the ROC analysis was 14.4 mg/l.

**Conclusion:**

Increasing IP vancomycin serum levels are associated with increased odds of cure; and maintaining vancomycin serum levels above 14–15 mg/l throughout the course of PD peritonitis treatment is likely to improve clinical outcomes.

## Graphical abstract

**Figure:**
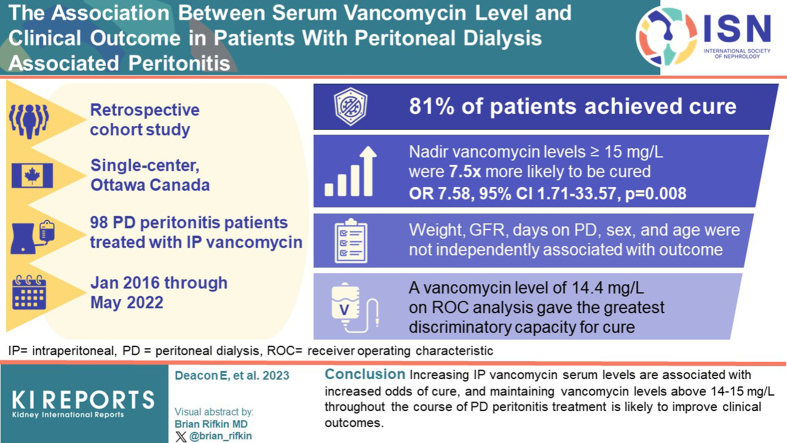

Peritonitis is a serious complication of PD and can lead to adverse outcomes, including the discontinuation of PD, peritoneal membrane damage, and death.^1^, ^2^, ^3^, ^4^ To mitigate the risk of these serious complications, empiric antimicrobial therapy is administered to patients treated with PD who manifest symptoms and signs of possible peritonitis. IP vancomycin is recommended as the first-line empiric therapy in centers with a high prevalence of methicillin-resistant gram-positive organisms and is then continued for susceptible organisms.^5^ After IP administration of vancomycin, 30% to 70% of the dose is absorbed into the systemic circulation following a 4-hour to 6-hour dwell.^6^ In subsequent antibiotic-free exchanges, peritoneal cavity concentration relies on back-diffusion of serum vancomycin into the peritoneal cavity.^6^ Trough serum levels have been shown to correlate with PD effluent concentration and are typically used as a proxy of vancomycin concentration in the peritoneal cavity.^7^

What constitutes an appropriate target serum vancomycin concentration in PD associated peritonitis is a source of debate. The 2016 International Society for Peritoneal Dialysis (ISPD) guidelines suggested that trough serum vancomycin levels should be maintained above 15 mg/l during treatment; however, there is no recommended target in the 2022 ISPD update.^1^^,^^2^ Current guidelines for intermittent vancomycin dosing leave much to the interpretation of the prescribing physician, suggesting 15 to 30 mg/kg every 5 to 7 days for continuous ambulatory PD, and 15 mg/kg every 4 days for automated PD. Therefore, vancomycin dosing protocols to treat PD peritonitis, both in terms of dosing and target serum levels, differ significantly between centers.^8^, ^9^, ^10^ We recently demonstrated that the patient’s weight and GFR affect the vancomycin trough serum level, and that consideration of these factors during dosing may help to maintain vancomycin levels above 15 mg/l.^11^ Fundamentally, however, the association between serum vancomycin and clinical outcomes, and how factors such as body composition and GFR may confound that relationship, remain unclear.

In this cohort of adult patients with PD peritonitis treated with IP vancomycin, our primary objective was to assess the likelihood of PD peritonitis cure in patients who had a nadir trough serum vancomycin level ≥15 mg/l versus those whose nadir level was <15 mg/l during treatment. The threshold of 15 mg/l was chosen based on the target level in the 2016 ISPD guidelines. Our secondary objective was to investigate if potential confounding patient factors, such as GFR and weight, affect the association between nadir vancomycin trough serum level and likelihood of cure. Finally, we explored whether alternative trough vancomycin serum cut-off levels could be more appropriate when considering the likelihood of cure.

## Methods

### Design, Setting, and Participants

This was a single-center retrospective cohort study of adult (>18 years old) patients who received IP vancomycin for the treatment of PD peritonitis diagnosed as per the ISPD guidelines at The Ottawa Hospital (Ontario, Canada) between January1, 2016 (when our program switched to empiric vancomycin) and May 31, 2022. PD peritonitis episodes were included if (i) patients completed a minimum 2-week course of IP vancomycin for a vancomycin-susceptible organism or culture-negative peritonitis, unless interrupted by an adverse outcome and (ii) at least 1 serum vancomycin level was measured. If patients had multiple PD peritonitis episodes during the collection period, their first episode meeting eligibility criteria was included. During the observation period, the standard empiric treatment for PD peritonitis included vancomycin 2 g IP (not weight-based) and either ceftazidime 1.5 g IP or a weight-based loading dose of tobramycin with adjustments made based on organisms isolated, sensitivities, and serum drug levels. For coagulase negative staphylococcus, culture negative peritonitis and susceptible enterococcus, single agent vancomycin was continued every 2 to 5 days aiming for a serum level of 15 to 25 mg/l.

Patients received 6-hour day dwells, and patients on automated PD were not switched to continuous ambulatory PD during peritonitis. Prior to June 2019, all relevant variables, including patient demographics, laboratory tests, and outcomes were collected from the Nephrocare (Fresenius Medical Care, Bad Homberg, Germany) clinical information system. Beyond June 2019, the same data were obtained from the patients’ electronic health records (Epic Systems, Verona, WI). The study was approved by the research ethics board of The Ottawa Hospital (approval number 20210456-01H).

### Variable Definitions

#### Exposure

The exposure of interest was the nadir vancomycin serum level. This was defined as the lowest recorded trough vancomycin serum level for each episode of PD peritonitis. The nadir level represents the time during treatment when theoretical peritoneal cavity concentration is at its lowest and patients may be at an increased risk of treatment failure and poor clinical outcomes. Based on previous ISPD guidelines,^12^ a threshold nadir vancomycin serum level of 15 mg/l was used for our primary analyses.

### Outcome

The primary outcome was a composite of all outcomes associated with peritonitis, as defined by the 2022 ISPD guidelines.^5^ As per these guidelines, cure was defined as complete resolution of peritonitis without relapse or recurrent peritonitis, catheter removal, transfer to hemodialysis for ≥30 days or death. Patients who later experienced repeat peritonitis greater than 4 weeks after completion of therapy of a prior episode with the same organism, or those who experienced hospitalization after initiation of vancomycin therapy but were ultimately cured and did not develop the aforementioned complications, were considered cured.

### Covariates

Patient demographic and clinical characteristics at the time of the index PD peritonitis episode that were collected included age, sex, PD modality (continuous ambulatory PD vs. automated PD), total number of days on PD, etiology of kidney failure, and microorganism associated with the peritonitis. GFR and weight data were collected from the PD adequacy assessment. In our program, an assessment of dialysis adequacy, including GFR is completed within 2 to 6 weeks of starting dialysis and then every 6 months. The weight and GFR were collected from the adequacy assessment that was completed most proximally to the peritonitis date.

### Statistical Analysis

The association between nadir vancomycin level and the primary outcome was evaluated in a logistic regression model, first unadjusted and then adjusted for potential confounding factors including age, sex, weight, GFR and total number of days on PD (per 100 days). Vancomycin was assessed both as a continuous exposure (per 1 mg/l increase) and as a categorical exposure (<15 mg/l vs. ≥15 mg/l). Estimates are reported as OR and associated 95% CIs. Output from all models used a complete case analysis with no imputation for missing data. An ROC curve was created to explore the sensitivity and specificity of various nadir vancomycin level thresholds (as a continuous measure) to identify an optimal vancomycin threshold. In a supporting analysis, the rates of cure and treatment failure were quantified across the distribution of nadir vancomycin level in increments of 1 mg/l. Continuous variables are reported as mean (SD) and categorical variables as count (percent). Days on PD is reported as median (interquartile range). Statistical analyses were performed using IBM SPSS Statistics 26.0 for Windows commercial software.^13^

## Results

### Patient Characteristics

**Figure 1:**
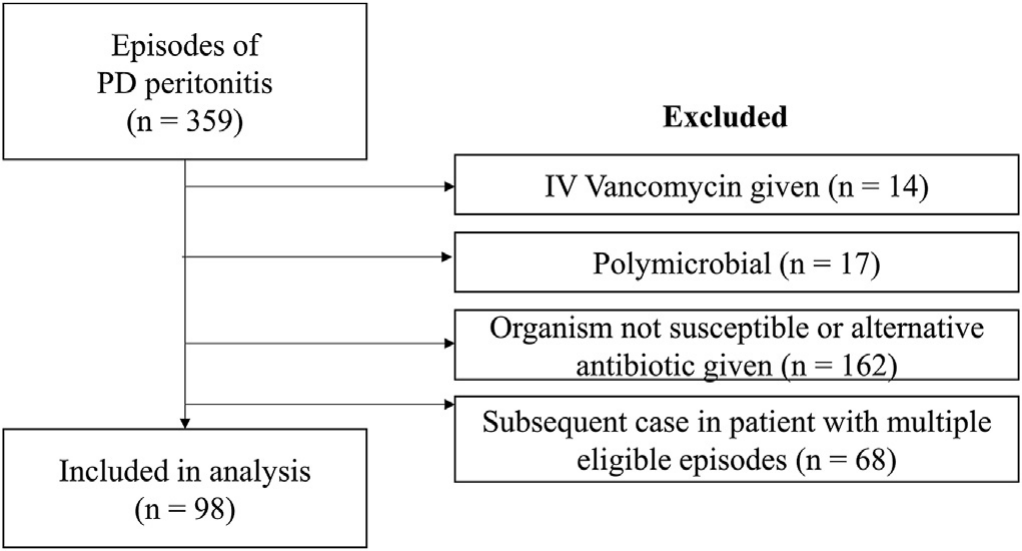
Flowchart of case ascertainment. PD, peritoneal dialysis.

**Figure 2:**
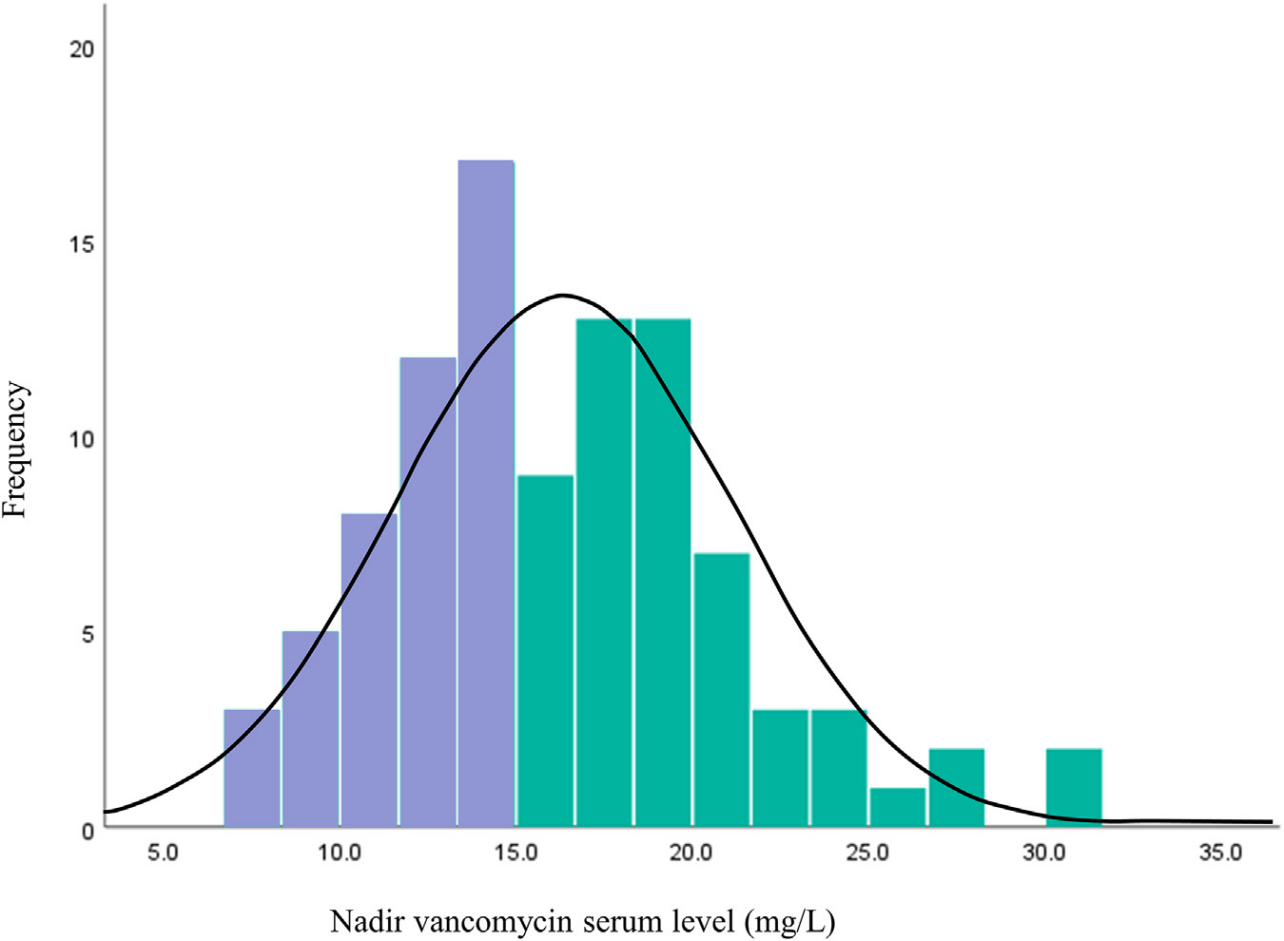
Distribution of nadir vancomycin serum levels. Values above 15 mg/l are shown in green; values less than 15 mg/l are shown in purple.

Out of 359 episodes of PD peritonitis between January 2016 and May 2022, we identified 98 patients who met the eligibility criteria (Figure 1). The distribution of serum nadir vancomycin concentration is shown in Figure 2. The median (interquartile range) number of days from the first day of peritonitis to nadir level drawn was 3 (3–4). As shown in Table 1, 54 patients had a nadir vancomycin serum level greater than or equal to 15 mg/l; and 44 patients had at least 1 value less than 15 mg/l. Among all included patients, the average age was 64 (±14) years, 72% were male and the most common etiology of kidney failure was diabetic nephropathy (45%). The mean GFR was 4.8 ml/min (±3.8) with approximately 40% of patients were being treated with continuous ambulatory PD. The median time spent on PD at the time of peritonitis diagnosis was 426 (±495) days and was right skewed, and the mean nadir vancomycin level was 16.4 (±4.8) mg/l. The average number of vancomycin levels measured was 2.3 and was similar between those who experienced cure (2.18) and those who did not (2.68). The most common cause of PD peritonitis was coagulase-negative staphylococci. Patient characteristics overall and stratified by nadir vancomycin level and cure status are shown in Table 1.

**Table 1:** Patient characteristics overall and stratified by outcome and nadir vancomycin serum level

Characteristics	All patients (*N* = 98)	Cure (*n* = 79)	No cure (*n* = 19)	Nadir level ≥15mg/l (*n* = 54)	Nadir level <15mg/l (*n* = 44)
Demographic Characteristics					
Male sex	71 (72%)	58 (73%)	13 (68%)	33 (61%)	38 (86%)
Age	64.4 (14.4)	63.9 (14.6)	66.5 (13.3)	62.4 (15.8)	66.8 (12.1)
Weight (kg)	78.8 (16.0)	78.5 (16.1)	80.0 (15.9)	76.5 (14.6)	81.5 (17.3)
Etiology of kidney disease					
Diabetic nephropathy	44 (45%)	38 (48%)	6 (32%)	24 (44%)	20 (46%)
Ischemic nephropathy	9 (9%)	7 (9%)	2 (11%)	5 (9%)	4 (9%)
Unknown	8 (8%)	5 (6%)	3 (16%)	3 (6%)	5 (11%)
Polycystic kidney disease	6 (6%)	4 (5%)	2 (11%)	3 (6%)	3 (7%)
IgA nephropathy	6 (6%)	6 (8%)	0 (0%)	5 (9%)	1 (2%)
Other	25 (26%)	19 (24%)	6 (32%)	14 (26%)	11 (25%)
GFR (ml/min)	4.8 (3.8)	4.6 (3.6)	5.4 (4.8)	3.90 (3.0)	5.8 (4.5)
PD modality					
CAPD	37 (38%)	27 (34%)	10 (52%)	21 (39%)	16 (36%)
APD	61 (62%)	52 (66%)	9 (47%)	33 (61%)	28 (64%)
Days on PD	426 (495)	405 (555)	484 (410)	426 (479)	433 (650)
Nadir vancomycin level (mg/l)	16.4 (4.8)	17.0 (4.9)	13.6 (3.3)	19.7 (3.6)	12.2 (2.1)
Number of levels drawn	2.3 (1.3)	2.18 (1.3)	2.68 (1.3)	2.43 (1.5)	2.09 (1.1)
Microorganism					
CNS	53 (54%)	46 (58%)	7 (37%)	30 (56%)	23 (52%)
No growth	22 (22%)	17 (22%)	5 (26%)	12 (22%)	10 (23%)
Enterococcus species	14 (14%)	9 (11%)	5 (26%)	8 (15%)	6 (14%)
Streptococcus species	6 (6%)	4 (5%)	2 (11%)	2 (4%)	4 (9%)
Coryneform	2 (2%)	2 (3%)	0 (0%)	1 (2%)	1 (2%)
No culture done	1 (1%)	1 (1%)	0 (0%)	1 (2%)	0 (0%)
Outcomes					
Cure	79 (81%)			50 (93%)	29 (66%)
Recurrent	8 (8%)			3 (6%)	5 (12%)
Relapsing	5 (5%)			1 (2%)	4 (9%)
Repeat	13 (13%)			4 (7%)	9 (21%)
Catheter removal	4 (4%)			0 (0%)	4 (9%)
Hemodialysis transfer	4 (4%)			0 (0%)	4 (9%)
Death	2 (2%)			0 (0%)	2 (5%)
Hospitalization	7 (7%)			2 (4%)	5 (12%)

APD, automated peritoneal dialysis; CAPD, continuous ambulatory peritoneal dialysis; CNS, coagulase-negative staphylococci; GFR, glomerular filtration rate. IgA, immunoglobulin A; PD, peritoneal dialysis.

Values are reported as mean (standard deviation) or count (percent).

## Outcomes

As shown in Table 1, a total of 79 patients (81%) experienced a cure of their PD peritonitis. Proportionally more patients with all vancomycin serum levels ≥15 mg/l achieved cure compared to those with at least 1 level <15mg/l (93% vs. 66%, *P* < 0.001). Seven patients (7%) were hospitalized as a result of their PD peritonitis. The most common complications among patients who did not achieve cure were recurrent (8%) and relapsing peritonitis (5%). Four patients (4%) required catheter removal with transfer to hemodialysis. There were 2 PD peritonitis-associated deaths.

## Association Between Nadir Vancomycin Level and Outcome

Patients with a nadir vancomycin level ≥15mg/l were approximately 5 times more likely to experience cure compared to patients with a nadir level <15 mg/l (OR 5.07, 95% CI 1.50–17.12, *P* = 0.009) (Table 2). This estimate was minimally attenuated with the addition of age to the model, whereas there was some evidence of negative confounding with the addition of sex to the model.

**Table 2:** Association between nadir vancomycin level and cure before and after adjusting for potential confounding variables

Nadir vancomycin level	GFR (per 1 ml/min)	Days on PD (per 100 d)	Weight (per kg)	Sex (women compared to men)	Age (Per yr)
(a) Nadir vancomycin level ≥ vs. < 15 mg/l					
5.07 (1.50–17.19)					
(*P* = 0.009)					
5.33 (1.53–18.55)	1.03 (0.89–1.19)				
(*P* = 0.009)	(*P* = 0.70)				
4.94 (1.45–16.89)		0.94 (0.84–1.05)			
(*P* = 0.01)		(*P* = 0.26)			
5.29 (1.54–18.19)			1.01 (0.97–1.04)		
(*P* = 0.008)			(*P* = 0.62)		
7.33 (1.82–29.52)				0.336 (0.09–1.48)	
(*P* = 0.005)				(*P* = 0.16)	
4.71 (1.38–16.11)					0.98 (0.94–1.03)
(*P* = 0.01)					(*P* = 0.39)
7.58 (1.71–33.57)	1.00 (0.86–1.17)	0.94 (0.84–1.05)	1.00 (0.96–1.04)	0.29 (0.06–1.40)	0.98 (0.93–1.03)
(*P* = 0.008)	(*P* = 0.96)	(*P* = 0.25)	(*P* = 0.81)	(*P* = 0.12)	(*P* = 0.98)
(b) Nadir vancomycin level (continuous, mg/l)					
1.17 (1.01–1.34)					
(*P* = 0.03)					
1.17 (1.01–1.36)	1.03 (0.88–1.19)				
(*P* = 0.032)	(*P* = 0.72)				
1.17 (1.01–1.35)		1.00 (0.99–1.00)			
(*P* = 0.03)		(*P* = 0.19)			
1.17 (1.02–1.34)			1.01 (0.97–1.04)		
(*P* = 0.03)			(*P* = 0.66)		
1.19 (1.03–1.39)				0.48 (0.13–1.75)	
(*P* = 0.02)				(*P* = 0.27)	
1.16 (1.01–1.33) (*P* = 0.04)					0.98 (0.93–1.02) (*P* = 0.33)
1.21 (1.02–1.43)	1.00 (0.86–1.17)	0.93 (0.84–1.04)	1.00 (0.96–1.04)	0.39 (0.09–1.67)	0.98 (0.93–1.03)
(*P* = 0.03)	(*P* = 0.97)	(*P* = 0.22)	(*P* = 0.83)	(*P* = 0.21)	(*P* = 0.35)

GFR, glomerular filtration rate; PD, peritoneal dialysis.

Estimates represent odds ratios (95% confidence intervals). (a) Nadir vancomycin level ≥15 mg/l versus <15 mg/l. (b) Nadir vancomycin level as a continuous measure (per 1 mg/l increase).

There were 3 episodes for which a GFR measurement was not available and 1 episode for which weight was not available. A complete case analysis (*N* = 94) was used for all models to facilitate comparison of estimates.

After adjusting for age, sex, weight, GFR, and days on PD (per 100 days), patients with nadir vancomycin level ≥15 mg/l were 7.5 times more likely to experience cure compared to patients with a nadir level <15 mg/l (OR 7.58, 95% CI 1.71–33.57, *P* = 0.008) (Table 2). GFR, weight, sex days on PD, and age were not independently associated with outcome. After adjusting for the same patient factors, each 1 mg/l increase in nadir vancomycin serum level was associated with 21% increased likelihood of cure (OR 1.21, 95% CI 1.02–1.43, *P* = 0.03) (Table 2).

A total of 4 patients were not included in the final multivariable model due to missing data for either GFR or weight. The characteristics of these 4 patients were similar to those of the 94 patients included in the model (Supplementary Table S1).

### Risk-Based Cut-Offs for Nadir Vancomycin Level

**Figure 3:**
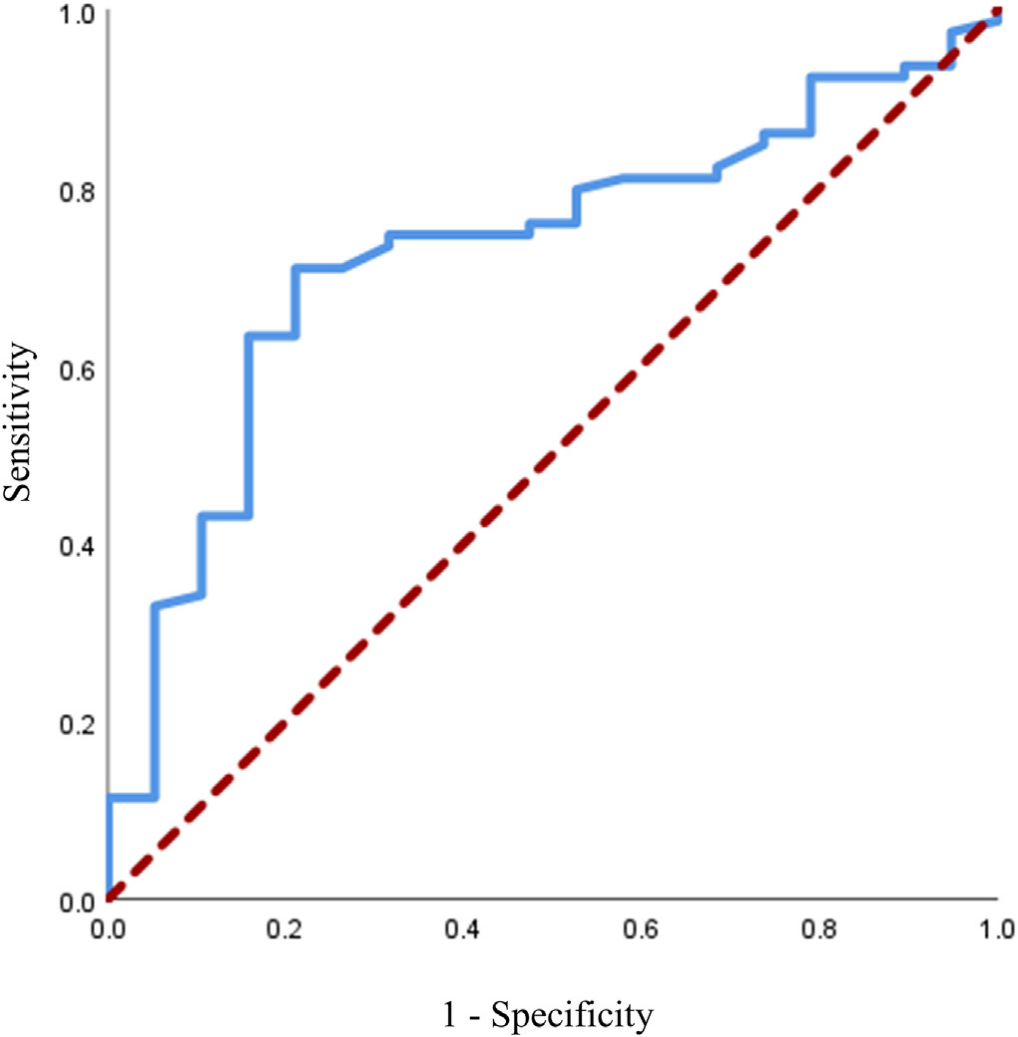
Receiver operating characteristic curve for nadir vancomycin serum levels as a continuous exposure. The model had an area under the curve of 0.73 (95% confidence interval 0.61 to 0.85).

In an attempt to identify an optimal cut-off for nadir vancomycin level, an ROC curve was generated from a model with continuous nadir vancomycin level as the sole explanatory variable (Figure 3). This model had an area under the curve of 0.73 (95% CI 0.61–0.85). The cut-off vancomycin level that optimized sensitivity and (1–specificity) was 14.4 mg/l. Rates of cure and noncure across a range of nadir vancomycin in increments of 1 mg/l are shown in Table 3. The largest difference between cure rate above and cure rate below occurred at the ≥14 mg/l and ≥15 mg/l nadir vancomycin serum level thresholds.

**Table 3:** Cure and fail rates for specific thresholds of nadir vancomycin concentration

Nadir Level (mg/l)	Cure rate above (confidence interval)	Cure rate below (confidence interval)	Total number episodes with nadir level ≥ indicated level	Total number episodes with nadir level < indicated level
≥10	0.81 (0.73, 0.89)	0.75 (0.64, 0.86)	90	8
≥11	0.83 (0.74, 0.91)	0.67 (0.59, 0.74)	86	12
≥12	0.83 (0.75, 0.91)	0.69 (0.63, 0.74)	82	16
≥13	0.84 (0.76, 0.92)	0.68 (0.64, 0.72)	76	22
≥14	0.89 (0.82, 0.97)	0.63 (0.60, 0.65)	66	32
≥15	0.93 (0.86, 1.00)	0.66 (0.64, 0.68)	54	44
≥16	0.94 (0.87, 1.00)	0.68 (0.66, 0.70)	48	50
≥17	0.93 (0.85, 1.00)	0.71 (0.70, 0.73)	42	56
≥18	0.94 (0.87, 1.00)	0.73 (0.72, 0.74)	35	63
≥19	0.96 (0.89, 1.00)	0.75 (0.73, 0.76)	27	71
≥20	0.94 (0.84, 1.00)	0.78 (0.76, 0.79)	18	80
≥21	0.93 (0.79, 1.00)	0.79 (0.78, 0.80)	14	84
≥22	0.91 (0.74, 1.00)	0.79 (0.78, 0.80)	11	87

## Discussion

In this single-center observational cohort study, we investigated the likelihood of PD peritonitis cure in patients with all trough serum vancomycin levels ≥15 mg/l during PD peritonitis treatment, versus those whose nadir level was <15 mg/l during treatment. In our adjusted analysis, we found that patients whose nadir vancomycin serum levels were maintained above 15mg/l throughout treatment were 7.5 times as likely to achieve cure compared to patients with any level less than 15 mg/l. The total number of days on PD, weight, age, sex, and GFR were not independently associated with clinical outcomes. Our exploratory analysis of alternative trough serum vancomycin thresholds identified 14.4 mg/l as the level with the greatest discriminatory capacity for cure. Overall, our novel investigation of the association between nadir vancomycin levels throughout the duration of PD peritonitis treatment and clinical outcomes supports that a target trough vancomycin serum level of 14 to 15 mg/l is correlated with an increased likelihood of achieving cure.

The association between nadir vancomycin serum levels and clinical outcomes during the treatment of PD peritonitis has varied between studies. Our results suggest that a vancomycin serum level <15 mg/l at any time point during PD peritonitis treatment decreases the odds of cure. Our results are largely in agreement with other retrospective cohort studies in which lower serum vancomycin levels were associated with a greater risk of relapse and adverse outcomes.^9^^,^^14^ However, our results are contrary to those of 2 other studies, but direct comparisons are difficult in light of differing definitions of outcomes, variables included in the models, patient populations examined, and the vancomycin serum levels that were directly assessed.^15^^,^^16^ The results of these studies are summarized in Table 4.

**Table 4:** Summary of retrospective cohort studies assessing association between vancomycin and clinical outcomes in PD peritonitis

Author	Yr	N	Exposure^a^	Outcome	Key findings	Limitations
Ma *et al.*^9^	2022	61 Gram-+or culture-negative episodes (in 46 patients)	Day 4 and day 2 IP vancomycin serum levels	Adverse outcomes (composite of transfer to hemodialysis, death, persistent infection, relapse)	Day 2 serum vancomycin levels were not predictive of adverse outcomes. Day 4 serum levels were significantly lower in the group with adverse outcomes (12.5 ± 4.3 mg/l vs. 8.4 ± 1.7 mg/l). ROC analysis of day 4 serum vancomycin levels identified 10.1 mg/l as the level with highest discriminatory capacity.	Single center, small sample size, all patients on CAPD limiting generalizability to patients on APD, non-independent observations (PD peritonitis episode to patient ratio >1:1)
Stevenson *et al.*^15^	2015	256 Gram-+ or culture-negative episodes (in 150 patients)	Day 2 IP vancomycin level ≥ 15 mg/l vs. < 15 mg/l, nadir day 2 vancomycin serum level	Cure (composite of any episode without relapse, catheter removal or death)	Day 2 serum vancomycin levels < 15 mg/l and those with day 2 levels ≥ 15 mg/l had similar rates of cure (60% vs. 69%, *P* = 0.69). Nadir serum vancomycin levels in the first week were not associated with cure (OR per mg/l 1.10, 95% CI 0.88–1.37, *P* = 0.39)	Single center, non-independent observations (PD peritonitis episode to patient ratio >1:1). Only vancomycin levels in the first week were considered.
Blunden *et al.*^16^	2007	449 Gram-+ or culture-negative episodes	Day 4 IP vancomycin serum level (where day of first dose = day 0)	Cure (resolution of peritonitis signs and symptoms where catheter was not removed)	Patients with day 4 level <12, 12–24 and >24 mg/l had respective cure rates of 77.9%, 74.2% and 75.0%, which were not statistically significantly different.	Single center, IP vancomycin dosing modified for weight and anuria limiting ability to assess these factors
Mulhern *et al.*^14^	1995	31 Gram-+ episodes (in 37 patients)	7-day trough IV vancomycin level	Relapse	Nine of 18 episodes with an initial 7-day trough level <12 mg/l had relapse; zero of 13 episodes with level >12 mg/l had relapse (*P* < 0.05).	Single center, small sample size, Gram-+ PD peritonitis only and IV vancomycin given limiting generalizability, non-independent observations (PD peritonitis episode to patient ratio >1:1)

APD, automated peritoneal dialysis; CAPD, continuous ambulatory peritoneal dialysis; CI, confidence interval; IP, intraperitoneal; IV, intravenous; OR, odds ratio; PD, peritoneal dialysis; ROC, receiver operating characteristic.

a Date of initial vancomycin dose defined as day = 0; study results adjusted as needed to aid in comparison.

We were unable to show a confounding effect of several patient factors, including GFR, weight, days on PD, and age, with the possible exception of sex. There is some evidence suggesting that reduced residual renal function is associated with poorer PD peritonitis-related outcomes, including mortality, possibly through mechanisms related to nutritional status.^17^^,^^18^ However, our results suggest that GFR is not independently correlated with the outcome but may indirectly impact outcomes through association with vancomycin serum levels. Although body mass index has been associated with an increased risk of PD peritonitis-related hospitalization, it is possible that heaver weight may be associated with lower serum vancomycin levels when dosing protocols are not weight-based and thus may indirectly impact outcomes.^11^^,^^19^ The peritoneal membrane undergoes extensive changes over time in patients treated with PD and time on dialysis has been a variable predictor of PD peritonitis outcomes.^20^, ^21^, ^22^ The numerical variability of time on PD was likely too small in our study to detect an association if one actually exists. Age and sex have been variably linked to PD peritonitis outcomes.^18^^,^^21^ Our results suggest that the male sex may be associated with increased likelihood of cure, however this finding was not statistically significant, which may indicate either a lack of association or an inability to detect one in the present patient sample. Vancomycin pharmacokinetics may be different in males and females with vancomycin distributed into excess body weight to a greater extent in women, potentially impacting peritoneal vancomycin levels.^23^

Although 15 mg/l has previously been suggested as a possible target threshold for trough vancomycin serum levels,^12^ we also sought to explore other possible cut-offs. Higher nadir vancomycin serum levels are likely to translate to higher vancomycin peritoneal effluent concentrations and thus theoretically higher cure rates; however, there are potential concerns about elevated vancomycin serum levels and nephrotoxicity.^24^ The level that optimized sensitivity and specificity of cure in this study was 14.4 mg/l. Ma *et al.*^9^ identified a lower optimal discriminatory capacity on ROC analysis (10.1 mg/l). In that study, 37% of patients were anuric, defined as 24-hour urine volume <100 ml. In our study population, almost all individuals had residual renal function. We recently demonstrated that reduced residual renal function may be associated with increased vancomycin serum levels.^11^ The vancomycin exposure-response relationship is often studied with a time-concentration curve, where both time and concentration are important when considering vancomycin efficacy.^25^ It is possible that lower target trough vancomycin serum concentrations may be appropriate in patients with anuria as in the study by Ma *et al.*,^9^ where the amount of time that vancomycin is above the minimum inhibitory concentration is theoretically increased.

Our study has some limitations, including a relatively small sample size contributing to wide error bounds for some estimates and possibly an inability to detect certain associations. To avoid overfitting the model, a select number of covariates were chosen and brought into the model sequentially to understand their potential confounding effect. Our study was based on a single center, thus limiting generalizability. Although patient covariates were based on the available measurements that were most proximal to the PD peritonitis episode, variables may still change over the PD time. We did not collect information on patients’ comorbidities or medication use, which may have contributed to unmeasured confounding. There was a relatively high rate of culture-negative peritonitis rate in our sample; however, cases were fairly evenly distributed between groups, so this is unlikely to bias our results. Owing to the retrospective study design, the frequency and timing of serum vancomycin levels were not standardized for all patients, which could have contributed to differences in outcomes. However, the average number of vancomycin levels drawn was similar among those who experienced cure and those who did not. Hospitalized patients represented a very small subgroup of the study population; therefore, we were unable to compare their outcomes to those managed in the outpatient setting. Our results cannot be extrapolated to PD peritonitis cases caused by methicillin-resistant Staphylococcus aureus because no cases were included in our sample.

## Conclusion

Maintaining trough vancomycin serum levels ≥15 mg/l was found to significantly increase the likelihood of cure during the treatment of PD peritonitis. Our program aims to maintain trough vancomycin serum levels between 15 and 25 mg/l with an initial 2 g IP vancomycin loading dose for most patients followed by subsequent drug level monitoring. However, in this study, 44 (45%) patients had at least 1 level that was less than 15 mg/l during treatment. We speculate that weight-based dosing may reduce the likelihood of underdosing of vancomycin during treatment to improve patient outcomes. Future studies assessing nadir vancomycin levels and clinical outcomes when a weight-based IP vancomycin dosing protocol is employed may improve our understanding of how to maintain adequate vancomycin serum levels during PD peritonitis and how to improve the likelihood of cure.

## Diclosure

All authors have declared no competing interests.

## Supplementary Material

Supplementary File (PDF)

**Table S1.** Characteristics of cases included in complete case analysis versus those not included.
