# Supplementary material for: The Association Between Serum Vancomycin Level and Clinical Outcome in Patients With Peritoneal Dialysis Associated Peritonitis
Source: Kidney Int Rep. 2023 Sep 17;8(12):2646–53. doi: 10.1016/j.ekir.2023.09.013 (PMC10719602; doi:10.1016/j.ekir.2023.09.013)
Supplement: Supplementary File (PDF) [file mmc1.pdf]

Supplementary Table S1: Characteristics of cases included in complete case analysis versus those not included

Characteristics	All patients (N=98)	Complete case (N=94)	Missing data (N=4)*
Demographic Characteristics			
Male sex	71 (72%)	68 (72%)	3 (75%)
Age	64.4 (14.4)	64.9 (14.3)	52.0 (11.2)
Weight (kg)	78.8 (16.0)	78.2 (15.7)	96.2 (15.9) *(N=3)
Etiology of kidney disease			
Diabetic nephropathy	44 (45%)	41 (44%)	3 (75%)
Ischemic nephropathy	9 (9%)	9 (10%)	0 (0%)
Unknown	8 (8%)	8 (9%)	0 (0%)
Polycystic kidney disease	6 (6%)	6 (6%)	0 (0%)
IgA nephropathy	6 (6%)	6 (6%)	0 (0%)
Other	25 (26%)	24 (26%)	1 (25%)
GFR (mL/min)[1]	4.8 (3.8)	4.7 (3.7)	12.9 *(N=1)
PD modality			
CAPD[1]	37 (38%)	37 (39%)	0 (0%)
APD[1]	61 (62%)	57 (58%)	4 (100%)
Days on PD	426 (495)	553 (451)	205 (257)
Nadir vancomycin level (mg/L)	16.4 (4.8)	16.5 (4.8)	12.6 (3.9)
Number of vancomycin levels measured	2.3 (1.3)	2.3 (1.4)	1.8 (0.5)
Microorganism			
CNS[1]	53 (54%)	52 (55%)	1 (25%)
No growth	22 (22%)	20 (21%)	2 (50%)
Enterococcus species	14 (14%)	13 (14%)	1 (25%)
Streptococcus species	6 (6%)	6 (6%)	0 (0%)
Coryneform	2 (2%)	2 (2%)	0 (0%)
No culture done	1 (1%)	1 (1%)	0 (0%)
Outcomes			
Cure	79 (81%)	78 (83%)	1 (25%)
Recurrent	8 (8%)	6 (6%)	2 (50%)
Relapsing	5 (5%)	4 (4%)	1 (25%)
Repeat	13 (13%)	13 (14%)	0 (0%)
Catheter removal	4 (4%)	4 (4%)	0 (0%)
Haemodialysis transfer	4 (4%)	4 (4%)	0 (0%)
Death	2 (2%)	2 (2%)	0 (0%)
Hospitalization	7 (7%)	7 (7%)	0 (0%)
